# Preventing Multimorbidity: Moving Beyond the Single Disease Lens

**DOI:** 10.3389/ijph.2025.1608880

**Published:** 2025-09-02

**Authors:** Tatjana T. Makovski, Sarah Cuschieri, Jose M. Valderas, Laurence Guldner, Iveta Nagyova, Marjan van den Akker, Valentina A. Andreeva, Michele Cecchini, Elio Riboli, Amaia Calderón-Larrañaga, Licia Iacoviello, Piotr Wilk, Laure Carcaillon-Bentata, Pierre Arwidson, Panayotis Constantinou, Fabrizio Faggiano, Kathryn Nicholson, Eric Breton, Franca Barbic, Julian Mamo, Laetitia Huiart, Joël Coste, Saverio Stranges

**Affiliations:** ^1^ Department of Non-Communicable Diseases and Injuries, French Public Health Agency (Santé publique France), Saint-Maurice, France; ^2^ Department of Anatomy, Faculty of Medicine and Surgery, University of Malta, Msida, Malta; ^3^ Department of Epidemiology and Biostatistics, Western University, London, ON, Canada; ^4^ Department of Medicine, National University of Singapore, Yong Loo Lin School of Medicine, Singapore, Singapore; ^5^ Department of Family Medicine, National University Health System, Singapore, Singapore; ^6^ Centre for Research in Health Systems Performance (CRiHSP), Yong Loo Lin School of Medicine, National University of Singapore, Singapore, Singapore; ^7^ Department of Social and Behavioural Medicine, Faculty of Medicine, PJ Safarik University, Kosice, Slovakia; ^8^ Institute of General Practice, Goethe-University Frankfurt, Frankfurt am Main, Germany; ^9^ Department of Family Medicine, Care and Public Health Research Institute, Maastricht University, Maastricht, Netherlands; ^10^ Department of Public Health and Primary Care, Academic Centre of General Practice, KU Leuven, Leuven, Belgium; ^11^ Nutritional Epidemiology Research Unit, Sorbonne Paris Nord University, INSERM/INRAE/CNAM, Epidemiology and Statistics Research Center (CRESS), Bobigny, France; ^12^ Health Division, Organisation for Economic Co-Operation and Development (OECD), Paris, France; ^13^ Cancer Epidemiology and Prevention Research Unit, School of Public Health, Imperial College London, London, United Kingdom; ^14^ Aging Research Center, Department of Neurobiology, Care Sciences and Society, Karolinska Institutet, Stockholm, Sweden; ^15^ Stockholm Gerontology Research Center, Stockholm, Sweden; ^16^ Research Unit of Epidemiology and Prevention, IRCCS Neuromed, Pozzilli, Italy; ^17^ Department of Medicine and Surgery, LUM University, Casamassima, Italy; ^18^ Department of Epidemiology and Population Studies, Institute of Public Health, Jagiellonian University, Kraków, Poland; ^19^ Department of Epidemiology, Maastricht University, Maastricht, Netherlands; ^20^ Bordeaux PharmacoEpi, INSERM CIC-P 1401, Université de Bordeaux, Bordeaux, France; ^21^ Department of Health Prevention, French Public Health Agency (Santé publique France), Saint-Maurice, France; ^22^ Direction of Strategy, Studies and Statistics, French National Health Insurance (Cnam), Paris, France; ^23^ Centre for Research in Epidemiology and Population Health, French National Institute of Health and Medical Research (INSERM U1018), Université, Paris‐Saclay, France; ^24^ Department of Sustainable Development and Ecological Transition, University of Piemonte Orientale, Vercelli, Italy; ^25^ Epidemiology Unit, ASL Vercelli, Vercelli, Italy; ^26^ Department of Family Medicine, McMaster University, Hamilton, ON, Canada; ^27^ Université de Rennes, EHESP and Arènes Research Unit (UMR CNRS 6051: Team INSERM U1309), Rennes, France; ^28^ Department of Biomedical Sciences, Humanitas University, Rozzano, Italy; ^29^ Internal Medicine, IRCCS Humanitas Research Hospital, Rozzano, Italy; ^30^ Department of Public Health, Faculty of Medicine and Surgery, University of Malta, Msida, Malta; ^31^ Scientific Directorate, French Public Health Agency (Santé Publique France), Saint-Maurice, France; ^32^ Department of Clinical Medicine and Surgery, University of Naples Federico II, Naples, Italy; ^33^ Department of Behavioural and Cognitive Sciences, University of Luxembourg, Esch-surAlzette, Luxembourg

**Keywords:** multimorbidity, multiple chronic conditions (MCCs), prevention, public health, policy

In September 2025 the Fourth High-level Meeting of the United Nations General Assembly (HLM4) will take place where the new vision to prevent and control non-communicable diseases (NCDs) towards 2030 and 2050 will be discussed. This will be an opportunity to evaluate current progress made to tackle chronic diseases worldwide. This commentary argues that it is time to add the multimorbidity prevention in the NCDs control policy agendas and it is supported by the ideas from the International Symposium on Multimorbidity, organised by Santé Publique France and the EUPHA Chronic Disease Section in June 2024 in Paris.

Due to global population ageing and overstretched healthcare systems, multimorbidity is now clearly established as a major public health concern, because of additional and potentially synergetic effects of each disease on health, and the secondary impacts of polypharmacy. A recent COVID-19 crisis also underscored a vulnerability of this population group in the face of that, and potentially other health threats in the future. Certain advancements were made, mostly in characterizing epidemiology of multimorbidity and its outcomes, and some on optimising healthcare, too. However, there is consensus that progress has been slow and insufficient, particularly in relation to the development of evidence-based prevention strategies.

The study by Feng at al [1] published in this journal underlines an increased risk of mortality for people with multimorbidity exposed to preventable risk factors such as abnormal sleeping time, smoking or drinking; while higher socio-economic status showed protective effects.

Previous work in multimorbidity prevention concentrated mostly on reducing, if not avoiding, further health deterioration in patients with multimorbidity, such as precluding further accumulation of diseases, decline in quality of life, and reducing unnecessary healthcare utilization and associated costs [2]. Primary prevention on multimorbidity is still only timidly present in the literature which can be explained partly by the challenges in implementing long-term interventions and long follow-up times that solid evidence to support this field may demand [3]. On the whole, more structure is sought to define prevention in the multimorbidity domain. Valderas (J.M.Valderas, personal communication, Jun 27, 2024) proposed a model distinguishing primary prevention, aimed at preventing transitions from 0 or one to 2 diseases; secondary prevention, minimising further accumulation of diseases; and tertiary prevention precluding occurrence of negative outcomes. Kuehlein et al. [4] completed this model with quaternary prevention arguing against unnecessary medical interventions as a core of “*primum non nocere*”, an aspect of particularly relevance for patients with multimorbidity who are more likely to experience duplication of diagnostic tests, polypharmacy and contradicting or abundant treatments compared with those living with single diseases due to multiple factors, including lack of compelling clinical guidelines, and challenges and gaps in communication between medical specialists and other healthcare providers.

Research for improved practice across all these domains is needed for advancing multimorbidity prevention. An additional difficulty is posed by different multimorbidity definitions, the different selection of eligible diseases, and the varying methodologies to identify the resulting combinations, what impedes exhaustiveness, precision and conclusiveness of the findings, in a sense of what patterns exactly to prevent [5]. Robust evidence would benefit from large longitudinal and repeated cross-sectional studies using standard diseases sets. From a public health perspective, these should include combinations of diseases with the highest frequency and/or impact, either at the individual level (e.g., quality of life) or at the system level (e.g., healthcare use and cost), as well as potential opportunities for prevention. Certain combinations with shared or associated etiology, such as cardiometabolic or musculoskeletal conditions, are well-known to clinical and public health practice, however these combinations are rarely surveyed or targeted for prevention together. Some other conditions such as anxiety, insomnia and eating disorders circulate frequently in the population, co-exist and show a good potential for prevention [6]. Moreover, multimorbidity profiles go beyond disease combinations and include socio-economic determinants and early-life experiences. Multimorbidity occurs earlier and is more prevalent among socially deprived groups resulting in different disease combinations such as those involving more mental health and functional limitations [7].

Effective individual preventive strategies for single conditions are available for reducing behavioural risk factors, i.e., smoking, alcohol or body mass index, as well as are population-level strategies, like policies that promote socially equitable and healthier environment and lifestyles [7]. This evidence is readily applicable to multiple conditions that share etiological pathways where a single action can be beneficial in preventing several diseases at once, hence in some instances even be referred to as a syndrome prevention approach. However, the underlying link between diseases is not always obvious, as evidenced by the different types of associations that have been recognized [8]: common risk factors; associated risk factors (correlated risks); direct causation between diseases; independence, when coexisting features of two conditions give rise to a third condition, and chance. A deeper understanding of these pathways would help predict and identify disease combinations, as well as inform targeted preventive strategies.

Preventive interventions can be difficult to design and implement due to the range of different multimorbidity profiles. However, as suggested by Head et al. [7], specifically including multimorbidity as an outcome in already existing interventions for individual chronic diseases would allow evaluating their effectiveness in preventing multiple diseases and/or subsequent diseases.

Interventions at the individual and group level should target high-risk populations, either early in life to foster healthy development or be tailored to different working conditions and/or later age groups, such as early retirement which may be associated with poor health outcomes. The integration of the primary care and public health is essential here considering a complexity of needs in patients with multimorbidity [9].

The recent review highlights the challenges to implement effective prevention interventions to reduce the burden of NCDs; almost half of countries are at risk of missing the goal to reduce NCD-related premature mortality by one-third [10]. The authors in addition propose interventions which promise to show results within 5-year timeframe trusting that the countries which lag behind will pick up on the actions to reach the set goals. And, while already existing difficulties to implement prevention strategies for individual diseases may seem further complicated with multimorbidity prevention, we think that it is time to introduce the term, including its primary, secondary, tertiary and quaternary niches, firmly in the policy agendas. Multimorbidity is no longer an issue of an older adults population only, neither a concern of an affluent parts of the world solely, therefore a global and lifelong approach to multimorbidity is required, as well as prevention of its consequences such as functionality and quality of life decline, and loss of autonomy. The management of patients with multimorbidity is inherently more complex, and health systems organized around single-disease models are often ill-suited to meet their needs. Investing in the prevention of multimorbidity could substantially alleviate the burden on healthcare systems. Consequently, it is essential to see beyond single diseases, and recognise potential of precluding occurrence of several conditions at once using common strategies where possible, to prevent and postpone those disease clusters amenable to prevention, ensuring longer and healthier lives and more sustainable healthcare and social systems.

While multimorbidity prevention did not find its place among 2030 Sustainable Development Goal targets, perhaps the upcoming UN General Assembly is a good opportunity for this. Raising awareness, developing and implementing prevention practices takes time, therefore the time should be given to multimorbidity prevention to assert its place in the public health agendas.
